# Confirmatory Factor Analysis of the French Version of the Savoring Beliefs Inventory

**DOI:** 10.3389/fpsyg.2018.00181

**Published:** 2018-02-19

**Authors:** Philippe Golay, Bénédicte Thonon, Alexandra Nguyen, Caroline Fankhauser, Jérôme Favrod

**Affiliations:** ^1^Community Psychiatry Service, Department of Psychiatry, University Hospital Centre, Lausanne, Switzerland; ^2^Psychology and Neuroscience of Cognition Research Unit, Department of Psychology, University of Liège, Liège, Belgium; ^3^La Source, School of Nursing Sciences, HES-SO University of Applied Sciences and Arts of Western Switzerland, Lausanne, Switzerland

**Keywords:** savoring, positive affect, emotion regulation, wellbeing, happiness

## Abstract

The Savoring Beliefs Inventory (SBI) is a measure designed to assess attitudes toward savoring positive experience within three temporal orientations: the past (reminiscence), the present moment (present enjoyment), and the future (anticipation). The aim of this study was to validate the structure of the SBI—French version. The scale was tested with 335 French-speaking participants. Two models were estimated: a one-factor model representing a general construct of savoring and a three-factor model differentiating between anticipation, present enjoyment, and reminiscence. Several indicators of model fit were used: the root mean square error of approximation (RMSEA), the comparison fit index (CFI), the Tucker–Lewis fit index (TLI), and the standardized root mean residual (SRMR). A chi-square difference test was used to compare the two models. The model fit of the three-factor model assessed by the SRMR showed to be excellent, while it could be considered as satisfactory according to the CFI and TLI coefficients. RMSEA, however, was slightly less adequate. The model fit for the one-factor model seemed less adequate than the three-factor solution. Further, the chi-square difference test revealed that the three-factor model had significantly better fit than the one-factor model. Finally, the reliability of the four scores (anticipating pleasure, present moment pleasure, reminiscing pleasure, and total score) was very good. These results show that the French version of the SBI is a valid and valuable scale to measure attitudes regarding the ability to savor positive experience, whether it be in anticipation, reminiscence, or the present moment.

## Introduction

Subjective wellbeing does not rely solely on the absence of distress, dysfunctional psychological processes, and mental disorders, nor on the ability to cope with negative experiences (Bryant and Veroff, 1984; Trompetter et al., 2017). The experience of positive emotions and, above all, the savoring of these pleasant emotions, have an independent and singular input for subjective wellbeing (Bryant, 1989; Carl et al., 2013; Hurley and Kwon, 2013). Savoring characterizes the ability to generate, increase, and prolong enjoyment, with a deliberate attentiveness to and awareness of the pleasure (Bryant, 2003; Jose et al., 2012). Facing the same positive event, two individuals will anticipate, enjoy, and reminisce to different extents and, therefore, experience different levels of positive emotions and wellbeing. Thus, it is not only the frequency of pleasant experiences or the ability to feel pleasure that matters to wellbeing but also the capacity to upregulate positive emotions. The ability to savor positive emotions has as much importance as dampening negative emotions has for subjective wellbeing (Nelis et al., 2011).

A large number of scales measure dysfunctional attitudes and emotional regulations (e.g., Garnefski and Kraaij, 2006; Innamorati et al., 2013). However, the exclusive use of these scales might not paint a reliable picture of one's emotional functioning, nor of one's subjective wellbeing (Nelis et al., 2011). A few measurements exist that capture one's ability to savor. Such scales would shed light on the strengths and limitations of an individual. Such evaluation would, consequently, guide therapy into relying on some emotional competencies and reinforcing or developing the weaker savoring abilities. Scales evaluating the ability to savor would also enable the evaluation of the effects of psychotherapy and any approach that intends to foster wellbeing, for example, interventions targeting emotional regulation and anhedonia (Meyer et al., 2012; Favrod et al., 2015).

To date, scales measuring positive emotion regulation in depth are limited to a few, including the Responses to Positive Affect scale (RPA) (Feldman et al., 2008), the Emotion Regulation Profile-Revised (ERP-R) scale (Nelis et al., 2011), and the Savoring Beliefs Inventory (SBI) (Bryant, 2003). The RPA focuses on the tendency to dampen positive emotions and to ruminate positively. Positive rumination consists of recurrently thinking of positive emotions or events (e.g., successes). The ERP-R measures several strategies related to emotion down-regulation and upregulation and includes both maladaptive and adaptive strategies. The adaptive positive emotion upregulation strategies include displaying positive emotions, mindfully savoring the present moment, capitalizing (i.e., celebrating and communicating about positive events), and positive mental time traveling (i.e., reminiscing or anticipating positive events). Other scales include only a few items that focus on positive emotion upregulation, such as the Emotion Regulation Questionnaire (Gross and John, 2003). The SBI was created to evaluate individuals' attitudes regarding savoring positive experiences. Its strength is its focus on positive upregulation of emotions and its inclusion of the three temporal orientations: the past (reminiscence), the present moment (present enjoyment), and the future (anticipation) (Bryant, 2003).

The SBI is composed of 24 items, each temporal orientation being represented by 8 items. Half of the items are positively formulated (e.g., “I find it easy to enjoy myself when I want to”), while the other half is negatively framed (e.g., “I don't like to look forward too much”). Thus, the scale measures, on the one hand, the propensity to savor pleasure and the beliefs in the capacity of savoring, and on the other hand, the negative attitudes concerning savoring and the difficulties one might have regarding the ability to savor.

The SBI has been validated in English-speaking populations (college students and elderly people) and shows good psychometric properties, as seen in the six studies conducted by Bryant (2003). Indeed, the total score of the SBI showed very good internal consistency (Cronbach's alpha between 0.88 and 0.94), and the subscales demonstrated moderate to high internal consistency (Cronbach's alpha between 0.68 and 0.89). Three-week test–retest correlations indicated strong temporal reliability (SBI total score, *r* = 0.84; Anticipating subscale, *r* = 0.80; Present moment subscale, *r* = 0.88; and Reminiscing subscale, *r* = 0.85, all *p* < 0.0001). The SBI total score correlated positively with various variables indicating good convergent validity, i.e., affect intensity (study 3, *r* = 0.48), optimism (study 4, *r* = 0.50), extraversion (study 4, *r* = 0.42), happiness intensity (study 3, *r* = 0.45; study 6, *r* = 0.56), percent of time happy (study 3, *r* = 0.55; study 6, *r* = 0.61), gratification (study 1, *r* = 0.39; study 2, *r* = 0.37), and self-esteem (study 1, *r* = 0.39; all *p* < 0.05, Bonferroni-adjusted). Good discriminant validity was evidenced by negative correlations between the SBI total score and hopelessness (study 4, *r* = −0.41), neuroticism (study 2, −0.38), physical anhedonia (study 4, *r* = −0.56), social anhedonia (study 4, *r* = −0.57), strain (study 2, *r* = −0.33), and percent of time unhappy (study 3, *r* = −0.35; study 6, *r* = −0.57; all *p* < 0.05, Bonferroni-adjusted).

Regarding gender differences, numerous studies have provided evidence that women experience joy and naturally savor pleasure to a greater extent than men do (e.g., Diener et al., 1999; Gentzler et al., 2016; but for a more complex review of the question, please refer to Zuckerman et al., 2017). Bryant found that women scored higher than did men on the SBI total scale [*F*_(1.445)_ = 11.21, *p* < 0.001], the Anticipating subscale [*F*_(1.445)_ = 9.18, *p* < 0.003], the Present moment subscale [*F*_(1.445)_ = 4.97, *p* < 0.03], and the Reminiscing subscale [*F*_(1.445)_ = 10.96, *p* < 0.001] (Bryant, 2003).

To date, there has not been any translation of the SBI into other languages. The goal of this study was to validate the French translation of the SBI and to determine which factor structure is more appropriate for the scale.

## Materials and methods

### Participants

Participants were 335 volunteers who were enrolled in the La Source School of Nursing Sciences in Lausanne as pre-graduate students or as professionals in continuous education courses (19.09% male and 80.91% female). The mean age was 28.09 years (*SD* = 9.72). Participants responded voluntarily and anonymously, there was no way they could be identified, and no personal data concerning their health were collected. They did not receive credit to participate. This study is outside the scope of the Swiss Human Research Act because no personal data concerning human diseases and concerning the structure and function of the human body (HRA art. 2) were collected. Therefore, this study did not need to be authorized by an ethics committee.

### Instrument

The SBI is a self-assessment questionnaire composed of 24 items, divided into three temporal orientations, past, present, and future, each represented by 8 items. Half of the items are positively formulated, while the other half are negatively framed. Each item is rated on a 7-point Likert scale ranging from “strongly disagree” to “strongly agree.” The total score of the SBI is calculated by subtracting the sum score of the negatively framed items from the sum score of positively phrased items. The three subscales—Anticipating pleasure, Present moment pleasure, and Reminiscing pleasure—are calculated in the same fashion. The Anticipating pleasure subscale measures savoring a future positive event beforehand, the Present moment pleasure subscale measures enjoying positive events when they occur and the Reminiscing pleasure subscale measures recalling past positive events after they have occurred.

The original English version of the SBI was independently translated by three native French-speaking members of our workgroup, JF, CF and AN, and compared until full agreement was found. The translation was authorized by the author of the original version.

### Statistical analyses

All reverse-scored items were re-coded before data-analysis. For the confirmatory factor analysis (CFA), item data were treated as categorical ordinal, and the models were estimated using a robust weighted least squares estimator with adjustments for the mean and variance (WLSMV). The hypothesized three-factor scoring structure was first tested (Bryant, 2003). It included an Anticipating pleasure factor (items 1, 4, 7, 10, 13, 16, 19, and 22), a Present moment pleasure factor (items 2, 5, 8, 11, 14, 17, 20, and 23), and a Reminiscing pleasure factor (items 3, 6, 9, 12, 15, 18, 21, and 24). Because a total score was also considered in the original scale, this model was compared to a more parsimonious structure including one general savoring factor.

Several indicators of model fit were used, such as the root mean square error of approximation (RMSEA), the comparison fit index (CFI), the Tucker–Lewis fit index (TLI), and the standardized root mean residual (SRMR). RMSEA < 0.06, SRMR < 0.08, and CFI/TLI > 0.95 are interpreted as having good fit, while values of RMSEA ≤ 0.08, SRMR < 0.10, and CFI/TLI ≥ 0.90 are considered as indicating acceptable fit (Hu and Bentler, 1999; Kline, 2005). One should note, however, that interpretation of global fit indexes in models with ordered categorical indicators is not as well established as it is with continuous indicators (Hu and Bentler, 1999). While simulation studies suggest that these cut-off values work reasonably well with categorical outcomes (Yu, 2002; Muthén, 2004), exact cut-off scores may not perfectly apply in the context of this study. Accordingly, alternative models were compared using a robust chi-square test using the DIFFTEST procedure. The reliability of the three subscales was estimated with McDonald's model-based Omega (ω) coefficient (Canivez, 2016). Age and gender differences were assessed by regressing each of the latent scores on the age and gender variables. All statistical analyses were performed with the Mplus statistical package version 7.4.

## Results

### CFA

**Figure 1:**
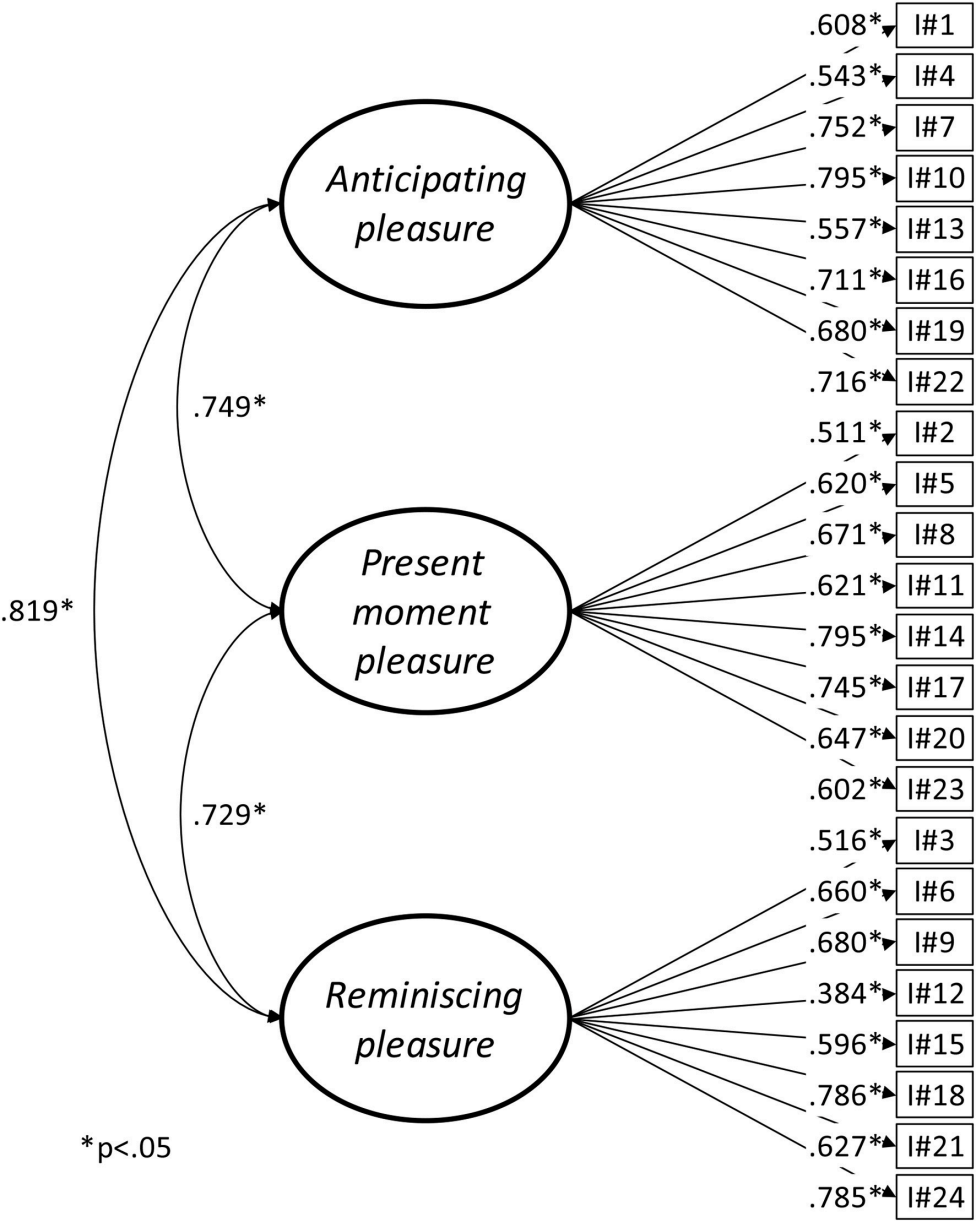
SBI three-factor model.

**Figure 2:**
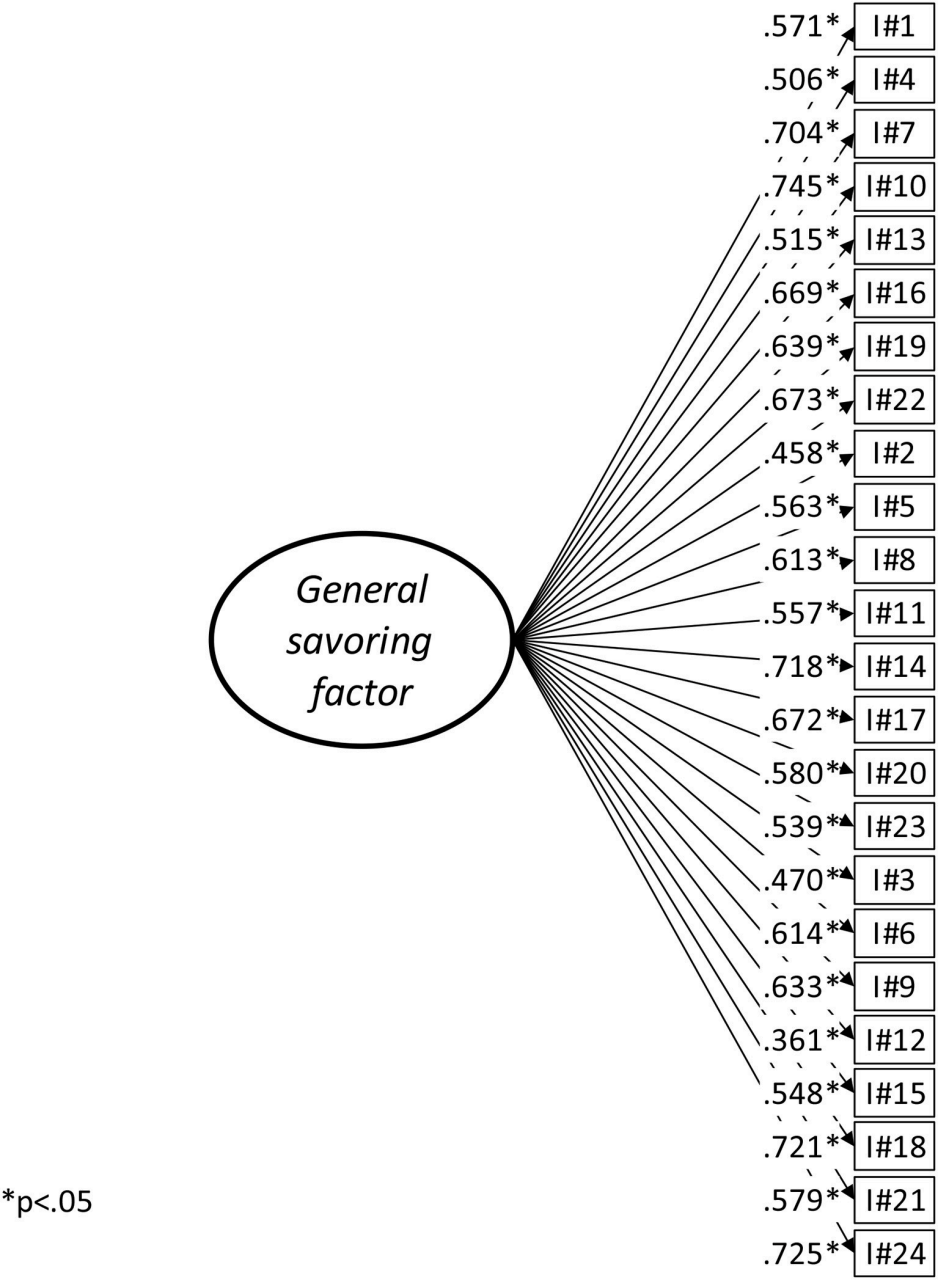
SBI one-factor model.

As shown in Table 1, the model fit of the three-factor model assessed by the SRMR was shown to be excellent, while it could be considered satisfactory according to the CFI and TLI coefficients. RMSEA, however, was slightly less adequate. Overall model fit could be considered as satisfactory and, as indicated on Figure 1, all factor loadings were supported. Factor correlations were high, suggesting that items could potentially be explained by a single dimension. A simpler one-factor model was estimated and compared to the three-factor structure. Model fit seemed less adequate than the three-factor solution although all factor loadings were supported (Figure 2). Because these two models were statistically nested, they could be compared using a robust chi-square difference test. Result confirmed that the three-factor model had significantly better fit than the one-factor model and should therefore be preferred (Δχ^2^ = 130.598, Δ*df* = 3, *p* < 0.001). Although statistically equivalent to the three-factor model, a higher-order model with three first-order factors loading onto a single overarching latent construct of savoring was estimated. The goal was to allow the determination of which of the three factors had the highest or lowest loading on the overarching construct. The loadings were high and quite similar (Anticipating pleasure = 0.917, Present moment pleasure = 0.817, Reminiscing pleasure = 0.893). The reliability of the four scores (ω Anticipating pleasure = 0.879, ω Present moment pleasure = 0.860, ω Reminiscing pleasure factor = 0.851, ω Total score = 0.941) was very good (Canivez, 2016). Additionally, when regressed on age and gender, the four latent scores (Total score, Anticipating pleasure, Present moment pleasure, and Reminiscing pleasure) were not significantly related with these socio-demographic variables (Table 2).

**Table 1:** Comparisons of model fit for the SBI (*N* = 335).

**Model**	***χ^2^***	***df***	***p*-value**	**RMSEA**	**CFI**	**TLI**	**SRMR**
Three-factor model	793.752	249	<0.001	0.081	0.915	0.905	0.069
One-factor model	1067.243	252	<0.001	0.098	0.872	0.860	0.082

*df, degree of freedom; RMSEA, Root Mean Square Error of Approximation; CFI, Comparative Fit Index; TLI, Tucker-Lewis Index; SRMR, Standardized Root Mean Square Residual*.

**Table 2:** Relationship between age and gender and the four factors.

	**Standardized Estimate**	**Standard Error**	***p*-value**
**ANTICIPATING PLEASURE**			
Gender	0.036	0.062	0.557
Age	−0.001	0.065	0.989
**PRESENT MOMENT PLEASURE**			
Gender	−0.021	0.064	0.738
Age	−0.019	0.067	0.779
**REMINISCING PLEASURE**			
Gender	0.122	0.064	0.055
Age	0.085	0.068	0.212
**GENERAL SAVORING FACTOR**			
Gender	0.047	0.060	0.435
Age	0.022	0.066	0.738

## Discussion

This study investigated the factor structure of the French version of the SBI. The results of the CFA indicated that the hypothesized three-factor structure of the French SBI was adequate, and all items contributed significantly to their corresponding factor: Anticipating pleasure, Present moment pleasure, and Reminiscing pleasure. The model-based reliability of all scores was very good. The three types of pleasure savoring were substantially correlated and shared between 53 and 67% of their variance. These results suggest that individuals able to experience pleasure in one of these three subdomains were more likely to be able to do so in the two other dimensions. However, based on the comparison between the one- and three-factor models, these three types of savoring may not be considered as undifferentiated and may represent theoretically meaningful and distinct dimensions. Despite the large amount of shared variance, there are theoretical benefits to conceptualizing savoring beliefs with three subscales rather than one (Bryant, 2003). The high correlation between the three subscales suggests that respondents are likely to have similar scores, on average. However, this will not always be the case, and, in our opinion, these differences may allow the identification of important clinical conditions.

Taken together, these results show that the SBI is a valid instrument to investigate savoring capacities in the three examined time frames.

In the original scale, compared to men, women showed higher mean scores on SBI total score and the different subscales (Bryant, 2003), which was not the case here. Further, in our sample, age was not related to any of the scores of the SBI.

Our study has several limitations that could be the focus of future studies. First, our sample comprised only students in the mental health field, which might not be representative of the general population in terms of savoring abilities. Further, our sample was relatively young and included a large majority of females. A study involving a more representative sample of the French-speaking population, including more males and elderly people, would further help understanding the different savoring abilities. Second, to further validate the French version of the SBI, concurrent and divergent validity must be examined. Further research on the psychometric characteristics of this scale may also include different clinical groups (e.g., people diagnosed with depression or schizophrenia). Finally, experimental designs may be used to examine the scale's sensitivity to change before and after psychosocial interventions.

The current study showed that the French version of the SBI is an internally valid instrument with very good model-based reliability. The results showed that the French version of the SBI was successfully adapted from the American version. This scale may, therefore, be a valuable tool for French-speaking clinicians and researchers who need to explore savoring attitudes, for instance, in relation to the maintenance or the development of wellbeing, as well as for the development of new interventions focusing on pleasure with clinical populations (Nguyen et al., 2016). The French version of the SBI completes the available scales for assessing pleasure in this language (Favrod et al., 2009; Chaix et al., 2017).

## Ethics statement

The project was conducted in accordance with ethical code regarding research with human participants and was exempted from the institutional review board.

## Author contributions

JF, PG, and AN designed this research. JF, AN, and CF independently translated the scale into French and found translation agreement. JF and AN acquired the data. PG and JF analyzed and interpreted the data. BT, PG, and JF drafted the first version of the manuscript. All the authors approved the final version for publication. All the authors agree to be accountable for all aspects of the work by ensuring that any questions related to its accuracy or integrity can be appropriately investigated and resolved.

### Conflict of interest statement

The authors declare that the research was conducted in the absence of any commercial or financial relationships that could be construed as a potential conflict of interest. The reviewer, RR, and handling Editor declared their shared affiliation.
